# The use of extruded finite-element models as a novel alternative to tomography-based models: a case study using early mammal jaws

**DOI:** 10.1098/rsif.2019.0674

**Published:** 2019-12-11

**Authors:** Nuria Melisa Morales-García, Thomas D. Burgess, Jennifer J. Hill, Pamela G. Gill, Emily J. Rayfield

**Affiliations:** 1 School of Earth Sciences, University of Bristol, Wills Memorial Building, Queens Road, Bristol BS8 1RJ, UK; 2 Smithsonian Institution, National Museum of Natural History, Washington, DC 20013-7012, USA; 3 Department of Earth Sciences, Natural History Museum, Cromwell Road, London SW7 5BD, UK

**Keywords:** *Morganucodon*, *Kuehneotherium*, finite-element analysis, stress, strain

## Abstract

Finite-element (FE) analysis has been used in palaeobiology to assess the mechanical performance of the jaw. It uses two types of models: tomography-based three-dimensional (3D) models (very accurate, not always accessible) and two-dimensional (2D) models (quick and easy to build, good for broad-scale studies, cannot obtain absolute stress and strain values). Here, we introduce extruded FE models, which provide fairly accurate mechanical performance results, while remaining low-cost, quick and easy to build. These are simplified 3D models built from lateral outlines of a relatively flat jaw and extruded to its average width. There are two types: extruded (flat mediolaterally) and enhanced extruded (accounts for width differences in the ascending ramus). Here, we compare mechanical performance values resulting from four types of FE models (i.e. tomography-based 3D, extruded, enhanced extruded and 2D) in *Morganucodon* and *Kuehneotherium*. In terms of absolute values, both types of extruded model perform well in comparison to the tomography-based 3D models, but enhanced extruded models perform better. In terms of overall patterns, all models produce similar results. Extruded FE models constitute a viable alternative to the use of tomography-based 3D models, particularly in relatively flat bones.

## 1. Background

Finite-element analysis (FEA) is an engineering technique that reconstructs stress, strain and deformation patterns in digital structures [1–3]. This method allows for a complex three-dimensional (3D) structure to be broken down into a finite number of elements of known material properties, size and shape whose response to a force can be readily quantified [1–3]. In vertebrates, FEA has mainly been used to assess feeding behaviour and mechanical performance of the skull in a wide array of groups, including cartilaginous fish [4], ray-finned fish [5], crocodilians [6], non-avian dinosaurs [7], birds [8], mammaliaforms [9], rodents [10,11], primates [12–14], bats [15], ungulates [16] and carnivorous mammals [17–20]. To a lesser extent it has been used in the study of locomotion and behaviour, for example, to assess the loading regime of the metatarsus in a theropod dinosaur [21], to study the mechanical potential of the manual ungual of dromaeosaurids in prey dispatching [22], to simulate sauropod trackway formation [23] and theropod dinosaur locomotion [24,25].

For its use in palaeontology, FEA has been validated using experimental approaches, including the *in vivo* analysis of primate [26] and American alligator skulls [27,28], *ex vivo* studies using a domestic pig cranium [16] and the mandible of an ostrich [8], and in macaque mandibles using *in vitro* data [29], as well as combined *in vivo* and *ex vivo* data [30]. Additionally, a comprehensive set of sensitivity analyses have been performed to improve finite-element (FE) models in terms of elastic properties and loading regimes [31–34], boundary conditions [29], mesh density [35] and generation [36], as well as element size and homogeneity [37].

In palaeontology, FE models are traditionally built using computed-tomography (CT) scan data. This method of data capture is widely used for FEA because it allows for the construction of very precise 3D models and because it captures the internal anatomy of the structures of interest [3]. Other approaches to 3D data capture, like photogrammetry, laser scanning and mechanical digitization, have been used to completely or partially build models for FEA [38–42] although these techniques are not able to capture internal anatomy. Alternatively, two-dimensional (2D) FE models (also known as planar models) have been used to study feeding biomechanics across the fish–tetrapod transition [43], analyse the skull mechanics of temnospondyls [44,45] and crocodilians [6], study the mechanical performance of dinosaur skulls [46–48] and cingulate xenarthrans [49], analyse the relationship between jaw shape and diet in primates [50] and assess the digestive physiology of ruminants using the robustness of their jaws [51], among others. It is simple, easy and quick to build 2D planar FE models and they represent a first approximation for performing large-scale studies and looking into general trends among clades [6,43,48,50]. Additionally, they do not require CT scan data, which can sometimes be inaccessible or very expensive. The simplicity of 2D planar models can be problematic, however, because they do not capture the three-dimensionality of the structure and the muscle configuration and the forces acting upon it, and must assume that the stresses and strains act only in the sagittal 2D plane [43]. It is, therefore, unclear to what extent 2D planar models can replicate the stress environment of a 3D shape. Until this relationship is assessed, the utility of 2D planar models and the potential for studying large-scale macroevolutionary trends cannot be fully realized.

Here, we test the utility of simple 2D planar FE models and simplified 3D models, to predict the stress response of a complex 3D structure. Recent 2D FE studies have focused on the vertebrate mandible, based on the assumption that it is a simple and largely planar structure that retains information about the feeding ecology of the individual. Here, we create simplified FE models of relatively flat mandibles, built digitally using a lateral 2D outline of the jaw and data on its mediolateral width. We focus on the Early Jurassic mammaliaforms *Morganucodon* and *Kuehneotherium,* two of the earliest and most basal representatives of the total group Mammalia*.* The biomechanical performance of the jaws of these taxa has been previously studied using 3D FE models built from CT scan data, alongside other biomechanical techniques [9]; therefore, they constitute ideal subjects for the validation of novel FE models. We create three types of FE model of increasing complexity: (a) 2D planar models, (b) *extruded* models, which have been extruded to the average width of the jaw and maintain a uniform thickness and (c) *enhanced extruded* models, similar to extruded models, but where the ascending ramus has been modified to more closely resemble the 3D geometry of the jaw. We compare stress and strain within the jaws of these simplified FE models to the complex 3D models to assess the utility of simplified approaches. Given that these models were built using fossil material, no *in vivo* validation was possible. Because these models represent isolated jaws only and the orientation of the adductor muscles cannot be accurately determined without a skull, we perform a series of sensitivity analyses to determine how the orientation of the muscle loads impacts the overall results when using the enhanced extruded models, as a means of helping us quantify uncertainty for incompletely preserved fossils.

## 2. Materials

We used the mandibles of two Early Jurassic (Hettangian–Early Sinemurian) stem mammals from Glamorgan, Wales, UK following Gill *et al.* [9]: *Morganucodon watsoni* (reconstructed from specimens UMZC Eo.D.61, UMZC Eo.D.45 (University Museum of Zoology in Cambridge, UK) and NHMUK PV M85507 (Natural History Museum, London, UK)) and *Kuehneotherium praecursoris* (reconstructed from specimens NHMUK PV M19766, NHMUK PV M19749, UMZC Sy.97 and NHMUK PV M92779). We used the FE models created by Gill *et al*. [9] as the basis for building 2D planar and extruded FE models and for comparison with the results from 3D FEA. The models in Gill *et al.* [9] are based on slightly incomplete specimens and the extruded models in this paper replicate this incomplete morphology.

## 3. Methods

### 3.1. Model creation

**Figure 1.**
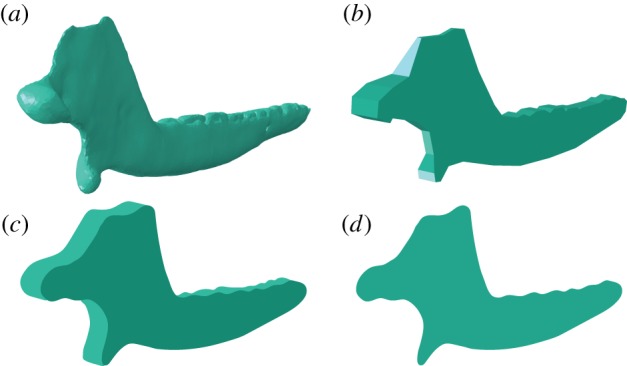
FE models analysed in this paper, using the example of *Morganucodon*: (*a*) CT scan-based 3D model, (*b*) enhanced extruded model, (*c*) extruded model and (*d*) 2D planar model. (Online version in colour.)

Examples of all the models used in this study are shown in figure 1 for *Morganucodon*. For *Kuehneotherium*, see electronic supplementary material, figure S1.

#### 3.1.1. Two-dimensional planar finite-element models

Lateral view screenshots of the mandibles were taken from fig. 1 in Gill *et al.* [9]. The mandibles were outlined in ImageJ v. 1.46r [52] using the multi-point feature. The resulting data were processed in Microsoft Excel to include only the XY coordinates of the outline. These data were imported into the computer-aided design (CAD) software Inventor Professional 2016 (Autodesk, USA) where a 2D model of the mandible was sketched using the spline function. The models (figure 1*d*) were then exported to a .STEP file for later use in the FEA software Abaqus v. 6.14–1 (SIMULIA, USA).

#### 3.1.2. Extruded finite-element models

2D planar FE models were constructed as above. These models were extruded medially to an average width in Inventor Professional 2016 (Autodesk, USA). The average width of the mandible was obtained from 10 equidistant measurements taken along the length of the mandible (in the 3D models used in Gill *et al*. [9]) in ImageJ v. 1.46r [52]. For *Morganucodon*, the length of the mandible was 21.11 mm and the average width was 0.81 mm. For *Kuehneotherium*, the length of the mandible was 20.76 mm (incisor region missing from original model) and the average width was 0.85 mm. The resulting extruded FE models (figure 1*c*) were exported to a .STEP file for later use in the FEA software Abaqus v. 6.14-1 (SIMULIA, USA).

#### 3.1.3. Enhanced extruded finite-element models

Alternative models to the simple, flat extruded FE models were generated. Using the 3D computer graphics software Blender v. 2.78, the mandibles were outlined and transformed into simple extruded models. Posteriorly, the ascending rami of the mandibles were modified in width to account for a more complex geometry (as shown in figure 1*b*) in three main areas: the coronoid process, the condyle and the angular process (i.e. those regions in which the lateromedial width of the ascending ramus was markedly different from that of the horizontal ramus). The region between the condyle and the top of the coronoid process, as well as the concavity of the angular, were likewise modified to obtain a gradual transition in width between areas. These structures were modified by taking additional width measurements from dorsal and posterior view screenshots of the 3D models of the jaws of *Morganucodon* and *Kuehneotherium* from Gill *et al*. [9] in ImageJ v. 1.46r [52]. These models were exported into .STL in Blender and converted to .STEP using the CAD software FreeCAD v. 0.16 for later use in the FEA software Abaqus.

### 3.2. Meshing

FEA requires models to be meshed into a finite number of elements of known size and shape. For all models, meshing was performed in the FEA software Abaqus v. 6.14-1 (SIMULIA, USA). As in Gill *et al*. [9], the mesh of extruded and enhanced extruded FE models used linear four-noded tetrahedral (C3D4) elements; the mesh of 2D planar models used three-node linear triangular (CPE3) elements. For a summary on the number of elements used in each mesh, see electronic supplementary material, table S1.

### 3.3. Finite-element analysis

#### 3.3.1. Material properties

Mandibles were assigned isotropic and homogeneous material properties of bone following Gill *et al.* [9], with Young's modulus of 18 GPa and Poisson's ratio of 0.3. As in Gill *et al*. [9], none of the models created included tooth crowns because edentate jaw models have been shown to perform better than dentate ones [29] and because the fossil specimens lacked some or all of the teeth. However, the models by Gill *et al*. [9] did include the tooth roots which had the material properties of dentine (i.e. Young's modulus of 25 GPa and Poisson's ratio of 0.3). In order to test whether the inclusion of dentine had a significant effect on the FEA results, the original Gill *et al*. [9] models were re-run with only one material (i.e. bone) in both mandible and tooth roots. The summary of the results can be found in electronic supplementary material, table S2. The stress, strain and reaction forces produced by the set of models with only the material properties of bone was almost identical to those produced by models with two different material properties (i.e. bone and dentine); therefore, the original models (with two material properties) do represent a good basis for validating the extruded models.

#### 3.3.2. Constraints and boundary conditions

Following Gill *et al*. [9], multi-point constraints with master (i.e. a single point representing the muscle attachment area in the absent skull in which the lines of action of the slave nodes converge) and slave nodes (i.e. a set of points that represent the muscle attachment area in the jaw) were applied at the mandibular condyle and at the biting point: m2 in *Morganucodon* and m3 in *Kuehneotherium*. There were approximately 32 slave nodes constrained at the condyle and 26 slave nodes constrained at the biting point in *Morganucodon* and approximately 23 slave nodes constrained at the condyle and 31 slave nodes constrained at the biting point in *Kuehneotherium* (muscle attachment regions across models encompass comparable areas but have slightly different number of nodes). Boundary conditions in all taxa were constrained in four degrees of freedom at the mandibular condyle (*U_1_* = *U_2_* = *U_R2_* = *U_R3_* = 0) and in four degrees of freedom at the biting point (*U_1_* = *U_2_* = *U_R2_* = *U_R3_* = 0). *U_1_* is the mesiodistal axis, *U_2_* is the dorsoventral axis and *U_3_* is the axis along the length of the jaw; *U* refers to translational movement, *U_R_* refers to rotational movement.

#### 3.3.3. Muscle attachment simulation

**Figure 2.**
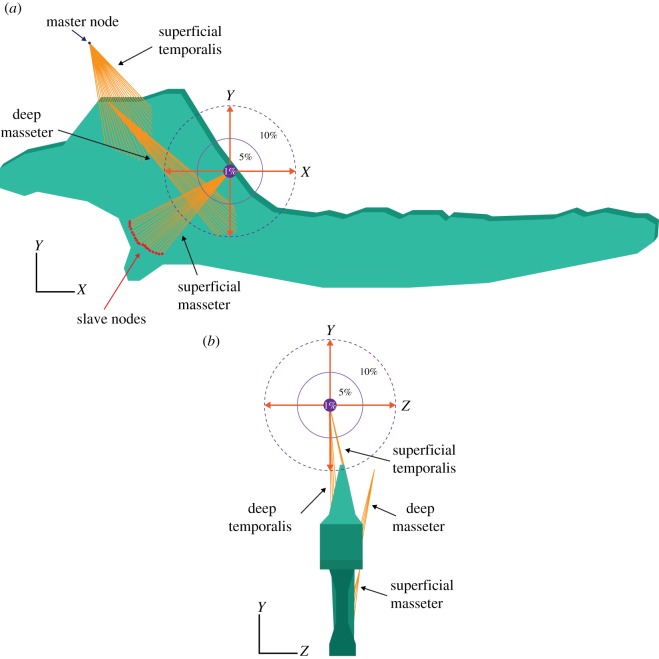
Sensitivity analyses. Jaw of *Morganucodon* in (*a*) lateral view and (*b*) posterior view depicting the range of distance (i.e. 1, 5 and 10% of the total length of the jaw) the muscles were moved during sensitivity analyses. (Online version in colour.)

For *Morganucodon* and *Kuehneotherium*, four muscles were modelled: superficial temporalis, deep temporalis, superficial masseter and deep masseter (figure 2). Multi-point constraints with master and slave nodes were used to simulate areas of muscle attachment at the mandible (slave nodes) and at the point they would attach to the skull (master nodes). Muscle loadings were different for each taxon and relative contributions of each muscle were calculated to obtain an overall bite force of 2 N in *Morganucodon* and 1.14 N in *Kuehneotherium*. The actual loading forces, obtained from Gill *et al*. [9], were as follows: superficial temporalis, 2 N; deep temporalis, 1.6 N; superficial masseter, 1.6 N; deep masseter, 1.6 N.

#### 3.3.4. Jaw performance

Reaction forces at the biting point and condyle were queried after running the model. Field output reports including maximum principal strain (i.e. tensile strain experienced by a bone following the application of a load [3]) and von Mises stress (i.e. parameter that predicts failure under ductile fracture [3]) were recorded for each model. Mesh-weighted arithmetic means (MWAM) were also calculated to account for differences in element size in the mesh following [37].

### 3.4. Sensitivity analyses

In order to evaluate the relevance of the accurate positioning of the master nodes of the muscle attachments (and the concomitant orientation of the muscle loads) in the absence of a skull, several sensitivity analyses were performed in the enhanced extruded FE models of *Morganucodon* and *Kuehneotherium*. These analyses involved moving the position of the master nodes of the temporalis, deep masseter and superficial masseter by 1%, 5% and 10% of the total jaw length in *x*, *y* and *z*, using a series of different transformation combinations (pictured in figure 2 and fully described in the electronic supplementary material). A total of 156 analyses were performed: 78 for *Morganucodon* and 78 for *Kuehneotherium*. The full compendium of the resulting stress and strain values obtained from these analyses can be found in the electronic supplementary material.

## 4. Results

### 4.1. Finite-element analysis

**Figure 3.**
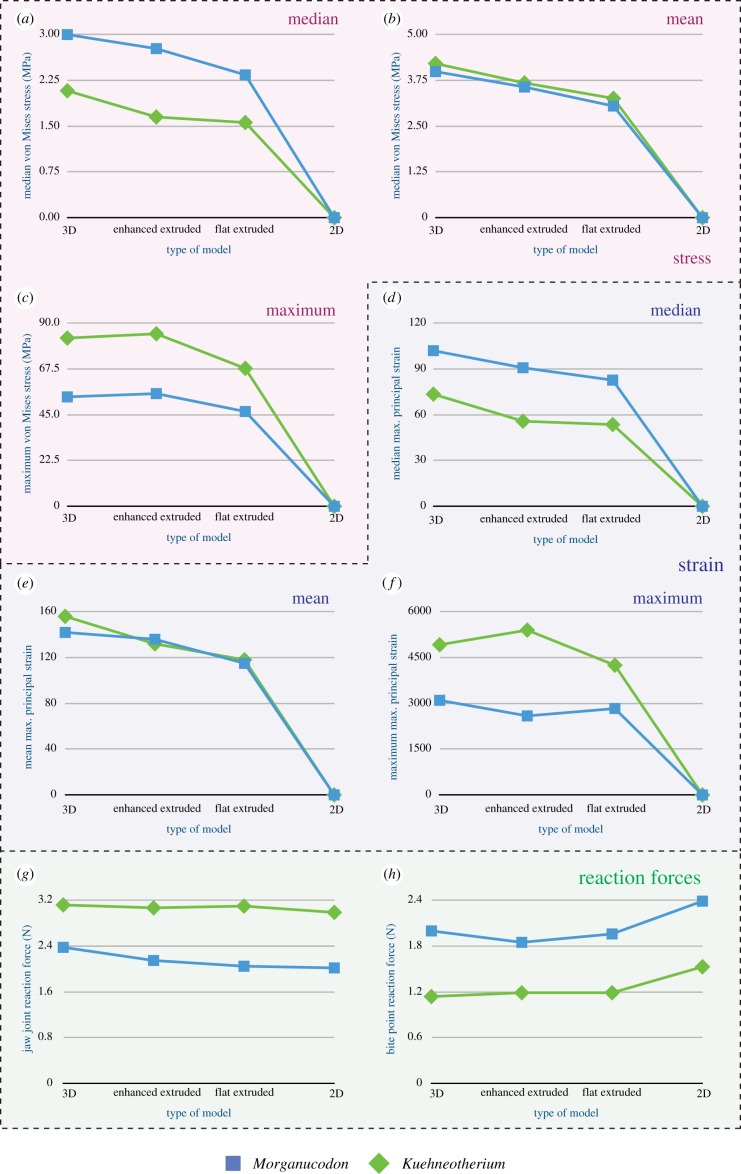
Comparative performance of the different FE models in terms of von Mises stress (MPa) (*a*: median, *b*: mean, *c*: maximum), maximum principal strain (*d*: median, *e*: mean, *f*: maximum) and reaction forces at the jaw joint (*g*) and bite point (*h*). *Morganucodon* in blue and *Kuehneotherium* in green. (Online version in colour.)

**Figure 4.**
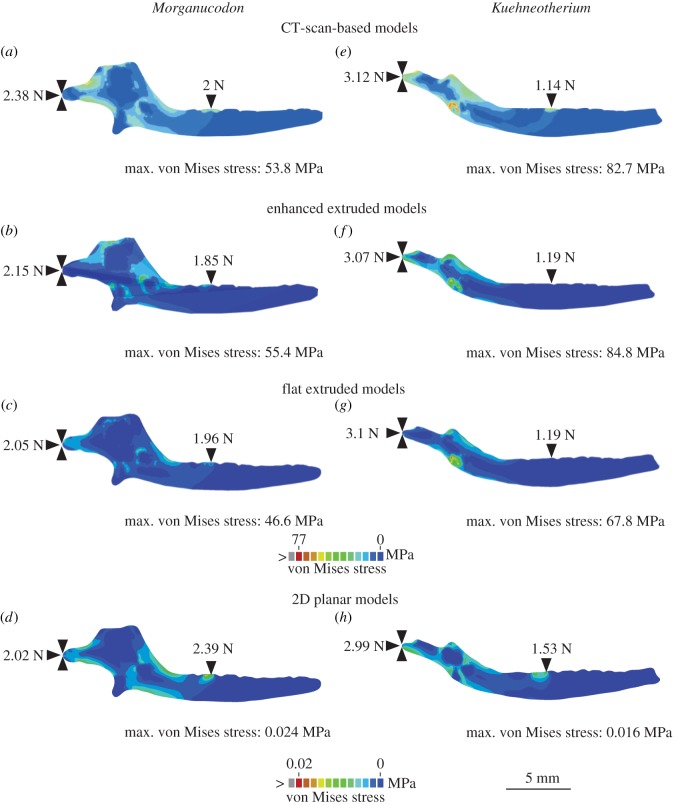
Finite-element stress plots of *Morganucodon* (*a–d*) and *Kuehneotherium* (*e–h*) using CT scan-based 3D models (*a,e*); extruded FE models: enhanced (*b,f*) and flat (*c,g*); and 2D models (*d,h*). Reaction forces, depicted by black triangles, shown for the jaw joint and the bite point (m2 in *Morganucodon* and m3 in *Kuehneotherium*). (Online version in colour.)

The comparative stress, strain and reaction forces of *Morganucodon* and *Kuehneotherium* obtained from FEA, using the four different models are summarized in table 1 and displayed as comparative plots in figure 3. Figure 4 shows the von Mises stress plots of all jaws. Particularly in table 1, the MWAM values calculated to account for element size differences in the mesh are fairly consistent with the arithmetic mean. This indicates that the size of the elements in the mesh is fairly homogeneous. Deformation patterns, fairly consistent across all models, are shown in electronic supplementary material, figure S2. In broad terms, the mean and median von Mises stress values resulting from both types of extruded FE models (enhanced and non-enhanced) were similar (75–92%) to those obtained from 3D models built from CT scan data. Particularly, enhanced extruded models produce more similar stress values to those obtained from the original 3D models, although they slightly overestimate (approx. 2.75%) the maximum stress experienced by the jaw. The mean and median von Mises stress values resulting from the 2D planar models were less than 0.05% similar to those obtained from 3D models built from CT scan data. Overall, the von Mises stress patterns in the jaws (figure 3) are fairly consistent across models, including the 2D planar models, with most of the stress being experienced around the muscle attachments and the biting point in both *Morganucodon* and *Kuehneotherium*. In figure 4*b,f*, this pattern is not evident in the enhanced extruded FE models because the von Mises stress scale was standardized for all 3D and extruded FE models, and the enhanced models experienced the lowest maximum stress values.

**Table 1.** Comparative results of biomechanical analyses: *Morganucodon* and *Kuehneotherium* under the four different FE models: A, CT scan-based 3D model; B, enhanced extruded model; C, extruded model; D, 2D planar model. MWAM, mesh-weighted arithmetic mean (following [15] and [37]). Green, more than 75% similarity with values obtained from 3D model; yellow, between 50 and 74%; red, less than 50% similarity.

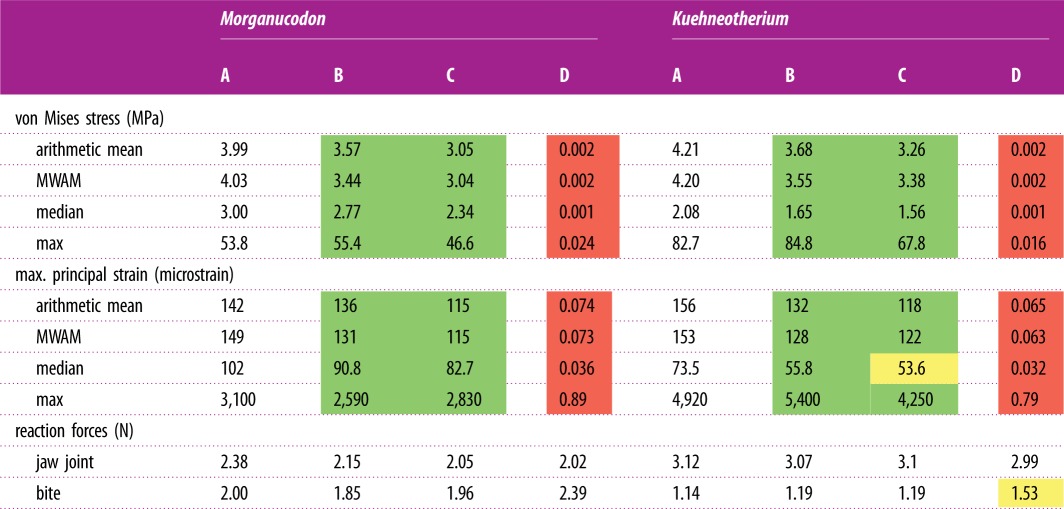

In terms of maximum strain magnitude, we obtained different results in both taxa. In the case of *Morganucodon*, the extruded FE model performed better than its enhanced counterpart, with the former achieving 91% of the original strain value and the latter only recovering 84%. By contrast, for *Kuehneotherium* the enhanced extruded FE model performed better than the simple extruded model, although it overestimated the maximum strain value by approximately 10%. In both taxa, the mean and median microstrain values were more similar between the original and enhanced extruded models, with the former recovering strain values of 90–96% of the 3D model in *Morganucodon* and 76–85% in *Kuehneotherium*. In both taxa, the mean, median and maximum strain values resulting from the 2D planar models were less than 0.05% similar to those obtained from 3D models built from CT scan data.

The reaction forces experienced at the jaw joint were similar across all FE models. In *Morganucodon*, the enhanced model recovered 90% of the original reaction force value, the flat extruded model recovered only 86%, and the 2D planar model was 85% similar. In the case of *Kuehneotherium*, both extruded FE models recovered approximately 99% of the original reaction force and the 2D model recovered 96%.

Both extruded FE models also performed well in terms of the reaction forces experienced at the biting point. In the case of *Morganucodon*, the flat extruded model produced a reaction force 98% similar to that produced by the original 3D model, while the reaction force produced by the enhanced extruded FE model was only 93% similar. For *Kuehneotherium*, the reaction forces in both models were identical, with both slightly overestimating the original value by approximately 4%. In the case of the 2D models, the reaction forces experienced at the biting point were overestimated in both models, by 20% in *Morganucodon* and 34% in *Kuehneotherium*.

### 4.2. Sensitivity analyses

**Figure 5.**
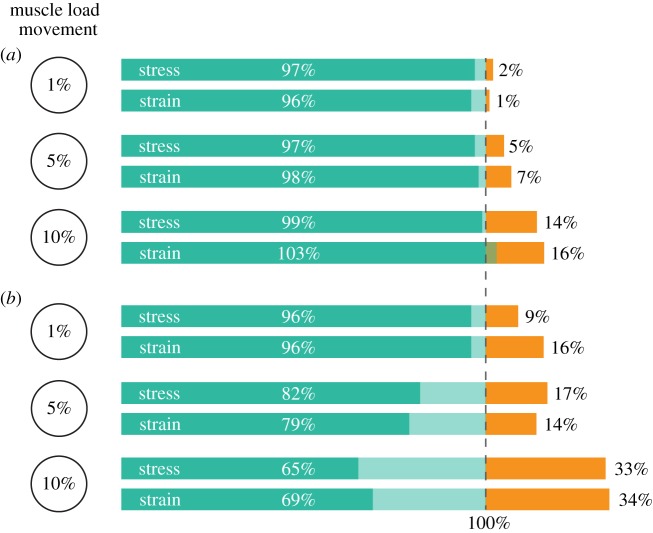
Results of the sensitivity analyses. Bar chart depicting the range of the mean and median stress and strain values observed in the sensitivity analyses for (*a*) *Morganucodon* and (*b*) *Kuehneotherium*. 100% line represents the original stress and strain results obtained from the enhanced extruded FE models, the green bar represents up to how much these results were underestimated in the sensitivity analyses, and the orange bar shows up to how much these results were overestimated. (Online version in colour.)

The summary of the results of the 156 sensitivity analyses, comparing the mean, median and maximum stress and strain values experienced in the enhanced extruded models with varying muscle positions to the original 3D models built from CT scan data, can be found in table 2. However, not all of these models depict a realistic orientation of the adductor muscles. Muscles were moved by a value determined as a percentage of jaw length. In some cases, moving muscles by 5% or 10% of jaw length resulted in muscle lines of action that were impossible; for example, passing through the ascending ramus of the jaw. In broad terms, the largest source of deviation from the original mean, median and maximum stress and strain values experienced by the jaw can be attributed to the unrealistic modelling of the adductor muscles, as can be seen when comparing table 2 (all iterations) with table 3 (only realistic muscle iterations). Overall, this unrealistic positioning is largely related to moving the muscle loads in the *z*-axis (figure 2*b*) past the anteroposterior axis of the jaw, effectively making the muscle pull in the opposite mediolateral direction of its natural orientation. Therefore, these models with unrealistic muscle orientations were removed, meaning that only 22 models were determined realistic for *Morganucodon* and 27 for *Kuehneotherium* (refer to electronic supplementary material for detailed results). These results are summarized in table 3 and graphically depicted in figure 5.

**Table 2.** Comparative stress and strain results of the sensitivity analyses—Includes: range (minimum and maximum values of all the iterations), standard deviation and % similarity range (i.e. percentage that represents the range of how much the stress and strain values deviated from the original results of the enhanced extruded FE models across all iterations of the sensitivity analyses).

	*Morganucodon*			*Kuehneotherium*		
	range (min–max)	s.d.	% similarity range	range (min–max)	s.d.	% similarity range
*moving all muscles 1%*						
von Mises stress (MPa)						
mean	3.5–3.8	0.1	97–106%	3.6–3.8	0.1	98–104%
median	2.7–2.9	0.04	99–104%	1.5–1.8	0.1	90–109%
max	54.7–56.1	0.4	99–101%	77.8–91.9	4.5	92–108%
maximum principal strain (microstrain)						
mean	131.3–146	5.3	96–107%	130.2–138.7	2.5	98–105%
median	90.1–96.2	2.04	99–106%	47.2–65.0	5.9	85–116%
max	2529.4–2968.2	121.01	98–115%	4952.9–5845	285.5	92–108%
*moving all muscles 5%*						
von Mises stress (MPa)						
mean	3.5–5.3	0.6	98–148%	3.5–5.6	0.7	95–153%
median	2.7–4.0	0.42	96–143%	1.4–4.0	0.9	82–246%
max	52–120	20	94–217%	65.4–121.1	17.4	77–143%
maximum principal strain (microstrain)						
mean	132.1–209.6	25.8	97–154%	125.9–214.9	29.6	95–162%
median	87.2–139.5	16.8	96–154%	43.9–151.2	35.5	79–271%
max	2760.7–5426.1	712.5	107–210%	3834.1–7794.5	1288.1	71–144%
*moving all muscles 10%*						
von Mises stress (MPa)						
mean	3.5–7.7	1.4	99–216%	3.5–8.8	1.8	95–238%
median	2.5–5.5	1.0	91–198%	1.1–6.8	2	65–414%
max	56.2–202.6	41.1	101–366%	63.8–187	32.8	75–220%
maximum principal strain (microstrain)						
mean	134–311.2	58.7	98–228%	125.8–343.6	75.4	95–259%
median	82.7–203.1	40.5	91–224%	38.7–259.8	74.4	69–465%
max	3108.3–8894.3	1560.9	120–343%	4064.2–11561.8	2026.5	75–214%

**Table 3.** Comparative stress and strain results of the sensitivity analyses with realistic muscle configurations—Includes: range (minimum and maximum values of all the iterations), standard deviation and % similarity range (i.e. percentage that represents the range of how much the stress and strain values deviated from the original results of the enhanced extruded FE models across all iterations of the sensitivity analyses).

	*Morganucodon*			*Kuehneotherium*		
	range (min–max)	s.d.	% similarity range	range (min–max)	s.d.	% similarity range
*moving all muscles 1%*						
von Mises stress (MPa)						
mean	3.5–3.6	0.1	97–101%	3.6–3.8	0.1	98–104%
median	2.7–2.8	0.02	99–102%	1.6–1.8	0.1	96–109%
max	54.7–55.7	0.3	99–101%	77.8–86.9	3	92–102%
maximum principal strain (microstrain)						
mean	131.3–137.7	2.5	96–101%	130–139	2.7	98–105%
median	90.1–92.1	0.7	99–101%	54–65	4	96–116%
max	2529.4–2735.8	66.8	98–106%	4952.9–5530.4	192.9	92–102%
*moving all muscles 5%*						
von Mises stress (MPa)						
mean	3.7–3.8	0.1	102–105%	3.6–4.1	0.2	97–112%
median	2.7–2.8	0.1	97–103%	1.4–1.9	0.3	82–117%
max	54.6–66	5	99–119%	75–99.7	9.8	88–117%
maximum principal strain (microstrain)						
mean	139.6–145.1	2.4	102–107%	127.8–150.9	8.6	96–114%
median	89.4–94.9	2.3	98–104%	43.9–62.5	8	79–112%
max	2985.1–3366.7	117.2	115–130%	4774.1–6065.8	623.6	88–112%
*moving all muscles 10%*						
von Mises stress (MPa)						
mean	3.78–4.06	0.12	106–114%	3.5–4.8	0.46	95–129%
median	2.74–3.02	0.11	99–109%	1.07–2.2	0.53	65–133%
max	58.87–92.91	14.09	106–168%	65.2–141.7	28.47	77–167%
maximum principal strain (microstrain)						
mean	145.8–157.9	5.46	106–116%	125.8–176.9	18.72	95–134%
median	93.23–102	3.85	103–113%	38.7–70.4	15.39	69–126%
max	3399.8–4305.9	415.52	131–166%	4152.7–8022.6	1410.94	77–149%

When considering only the models with realistic muscle orientations (figure 5) it is apparent that, in broad terms, the more the muscle loads are moved from their original position, the more the resulting stress and strain values deviate from the original values (i.e. those of the enhanced extruded FE models depicted in table 1). However, in all cases, these values proportionally deviate more in *Kuehneotherium* than in *Morganucodon*. Additionally, the mean and median stress and strain values tend to change fairly consistently throughout iterations, but the maximum values, particularly when moving the muscles over 5% of the total length of the jaw, deviate considerably (table 3).

In *Morganucodon*, the mean and median stress and strain values resulting from the 1% muscle movement sensitivity analyses did not deviate more than 4% from the original values obtained from the enhanced extruded model. The 5% movement sensitivity analyses results did not deviate more than 7% and the 10% movement sensitivity analyses did not deviate by more than 16%. The sensitivity analyses models tended to overestimate the mean and median stress and strain values in this taxon.

In *Kuehneotherium*, the mean and median stress and strain values resulting from the 1% muscle movement sensitivity analyses deviated up to 16% from the original values obtained from the enhanced extruded model. The 5% movement sensitivity analyses results deviated up to 21% and the 10% movement sensitivity analyses deviated up to 34%. The sensitivity analyses models tended to both underestimate and overestimate the mean and median stress and strain values in this taxon.

## 5. Discussion and conclusion

The more the geometric configuration of the digitally built models resembles that of the most accurate 3D digital representation of the jaw (i.e. the 3D models built from CT scan data), the closer their stress and strain values. In descending order of similarity these are (a) the enhanced extruded FE models, (b) the extruded FE models, and finally (c) the 2D planar FE models. Both types of extruded FE models produced results which, in most cases, recovered more than 75% of the stress and strain values observed in the original 3D models. However, 2D planar FE models achieved less than 0.05% of these values. Regardless, the von Mises stress plots across all models show fairly similar patterns, and the reaction forces in the jaw joint and biting point are closely comparable in most cases, including in the 2D models.

2D planar FE models are a popular alternative to the use of 3D models built from CT scan data because of their efficiency when performing large-scale studies and because they are valuable as a first approximation to evaluate the overall von Mises stress patterns experienced in the jaw [6,43,48]. However, as previously mentioned by these authors and further demonstrated here, 2D planar models cannot replicate the absolute stress and strain magnitudes experienced by the jaw because they represent an oversimplification of the geometry of the jaw and of the line of action of the adductor muscles. 2D models can, however, represent reaction forces and comparative patterns of stress and strain, presumably so long as muscle lines of action do not deviate far from the 2D plane of the model (although this remains to be tested). The use of extruded FE models can better approximate the 3D geometry of the jaw and its muscle configuration and produce similar stress and strain values to those obtained from 3D models built with CT scan data, while still preserving the economy and efficiency of 2D models. In terms of replicating absolute stress and strain magnitudes, enhanced extruded FE models constitute one of the best alternatives to the use of 3D models when no CT scan or photogrammetry data are available. Extruded FE models also constitute a viable alternative because they are easier and quicker to build than their enhanced counterparts, while still producing similar stress and strain values. Similar results have been obtained from Rahman and Lautenschlager's box models [53] in FEA using a skull of *Allosaurus* and a vertebra of *Stegosaurus*. Their models, which also represent a 3D simplification of the geometry of bone, have been assessed qualitatively (e.g. von Mises stress plots) and quantitatively (e.g. stress, strain, deformation) and perform in a similar manner to extruded models.

A large number of FEAs evaluating the mechanical performance of the jaw have been performed without the cranium (e.g. [9,28,29,37,43,48,49,51]). Given that both types of extruded FE models presented here are only built for the jaw and not the cranium, we cannot be certain we are realistically modelling muscle lines of action. Therefore, sensitivity analyses were performed in the enhanced extruded models to evaluate how much the resulting stress and strain values would change if the muscle loads were moved in various directions. As previously mentioned, the unrealistic modelling of the muscle loads in the *z*-direction was the largest source of deviation from the original stress and strain values, as well as moving the muscle loads by more than 10% of the total length of the jaw in both *Morganucodon* and *Kuehneotherium*; therefore, the understanding of how the adductor muscles attach to the cranium should be as thorough as possible.

The stress and strain values resulting from both types of extruded FE models represent a close approximation to the results obtained from 3D FE models built from CT scan data in relatively flat bones, particularly jaws. These models are still subject to the same assumptions as any other biological FE model, in terms of estimating material properties and boundary conditions such as muscle loads and constraints. The economy and efficiency with which they can be both built and analysed, while still providing reliable approximations of the stress and strain magnitudes experienced in the jaw, makes them a good alternative to the use of 2D planar models when performing large-scale studies where questions of comparative shape performance are warranted. Given the nature of how these models are built, reconstructions based upon a number of incomplete specimens are possible in a relatively easy manner, which is advantageous when dealing with fossil material. The use of early mammal jaws for building extruded FE models has proven useful since they are relatively flat and lack considerable anterior or other curvature along their length. How more three-dimensionally complex jaws may lend themselves to the extruded approach deserves further attention. Likewise, and to explore the full potential of this method, further studies can be made on the validation of extruded FE models on different morphologies (e.g. skull, limb bones, etc.). Enhanced extruded models, which provide more accurate results than simple extruded models, can be made as geometrically complex as needed; however, this can be a time-consuming process and could generate problems with meshing (further validation is needed). Other tools, like photogrammetry, can be performed at low cost to obtain 3D structure; however, other factors, like the size of the specimen, can present considerable obstacles to this technique. Additionally, the presence of an obscuring matrix around the fossil can be challenging for both photogrammetry and for building extruded models. While extruded models can be built from the reconstruction of several specimens, it is important to understand the limitations of the technique (e.g. must have a dorsal view picture to accurately estimate width of the jaw). On the other hand, enhanced extruded FE models are advantageous because they can be built using only a reduced number of pictures (i.e. lateral view, dorsal view, posterior view and, optionally, ventral view) as opposed to photogrammetry. Enhanced or simple extruded FE models, therefore, offer an alternative to 2D planar and CT scan-based 3D models for representing the mechanical behaviour of relatively flat geometric structures, such as the mammalian mandible.

## Supplementary Material

Supplementary Information

## Data accessibility

The FE models (CAE) from Abaqus and the field output reports are stored in https://www.doi.org/10.5523/bris.2b5w9l61qc6vl2rrkhv0sdex6v.

## References

[1] Zienkiewicz OC 1971 The finite element method in engineering science. New York, NY: McGraw-Hill.

[2] Zienkiewicz OC, Taylor RL 2000 The finite element method. Volume I: the basis. Barcelona, Spain: Butterworth-Heinemann.

[3] Rayfield EJ 2007 Finite element analysis and understanding the biomechanics and evolution of living and fossil organisms. Annu. Rev. Earth Planet. Sci. 35, 541–576. (10.1146/annurev.earth.35.031306.140104)

[4] Wroe S et al. 2008 Three-dimensional computer analysis of white shark jaw mechanics: how hard can a great white bite? J. Zool. 276, 336–342. (10.1111/j.1469-7998.2008.00494.x)

[5] Grubich JR, Huskey S, Crofts S, Orti G, Porto J 2012 Mega-Bites: extreme jaw forces of living and extinct piranhas (Serrasalmidae). Sci. Rep. 2, 1009 (10.1038/srep01009) 23259047 PMC3526859

[6] Pierce SE, Angielczyk KD, Rayfield EJ 2008 Patterns of morphospace occupation and mechanical performance in extant crocodilian skulls: a combined geometric morphometric and finite element modeling approach. J. Morphol. 269, 840–864. (10.1002/jmor.10627) 18496856

[7] Rayfield EJ, Norman DB, Horner CC, Horner JR, Smith PM, Thomason JJ, Upchurch P 2001 Cranial design and function in a large theropod dinosaur. Nature 409, 1033–1037. (10.1038/35059070) 11234010

[8] Cuff AR, Bright JA, Rayfield EJ 2015 Validation experiments on finite element models of an ostrich (*Struthio camelus*) cranium. PeerJ 3, e1294 (10.7717/peerj.1294) 26500813 PMC4614885

[9] Gill PG, Purnell MA, Crumpton N, Brown KR, Gostling NJ, Stampanoni M, Rayfield EJ 2014 Dietary specializations and diversity in feeding ecology of the earliest stem mammals. Nature 512, 303–305. (10.1038/nature13622) 25143112

[10] Cox PG, Rayfield EJ, Fagan MJ, Herrel A, Pataky TC, Jeffery N 2012 Functional evolution of the feeding system in rodents. PLoS ONE 7, e36299 (10.1371/journal.pone.0036299) 22558427 PMC3338682

[11] Cox PG, Rinderknecht A, Blanco RE 2015 Predicting bite force and cranial biomechanics in the largest fossil rodent using finite element analysis. J. Anat. 226, 215–223. (10.1111/joa.12282) 25652795 PMC4337660

[12] Strait DS et al. 2009 The feeding biomechanics and dietary ecology of *Australopithecus africanus*. Proc. Natl Acad. Sci. USA 106, 2124–2129. (10.1073/pnas.0808730106) 19188607 PMC2650119

[13] Tseng ZJ, Binder WJ 2010 Mandibular biomechanics of *Crocuta crocuta, Canis lupus*, and the late Miocene *Dinocrocuta gigantea* (Carnivora, Mammalia). Zool. J. Linn. Soc. Lond. 158, 683–696. (10.1111/j.1096-3642.2009.00555.x)

[14] Dumont ER, Davis JL, Grosse IR, Burrows AM 2011 Finite element analysis of performance in the skulls of marmosets and tamarins. J. Anat. 218, 151–162. (10.1111/j.1469-7580.2010.01247.x) 20572898 PMC3039787

[15] Dumont ER, Piccirillo J, Grosse IR 2005 Finite-element analysis of biting behavior and bone stress in the facial skeletons of bats. Anat. Rec. Part A 283, 319–330. (10.1002/ar.a.20165) 15747350

[16] Bright JA, Rayfield EJ 2011 Sensitivity and ex vivo validation of finite element models of the domestic pig cranium. J. Anat. 219, 456–471. (10.1111/j.1469-7580.2011.01408.x) 21718316 PMC3196751

[17] Wroe S, Clausen P, McHenry C, Moreno K, Cunningham E 2007 Computer simulation of feeding behaviour in the thylacine and dingo as a novel test for convergence and niche overlap. Proc. R. Soc. B 274, 2819–2828. (10.1098/rspb.2007.0906) PMC2288692 17785272

[18] Wroe S 2008 Cranial mechanics compared in extinct marsupial and extant African lions using a finite-element approach. J. Zool. 274, 322–339. (10.1111/j.1469-7998.2007.00389.x)

[19] Slater GJ, Figueirido B, Louis L, Yang P, Van Valkenburgh B. 2010 Biomechanical consequences of rapid evolution in the polar bear lineage. PLoS ONE 5, e13870 (10.1371/journal.pone.0013870) 21079768 PMC2974639

[20] Oldfield CC, McHenry CR, Clausen PD, Chamoli U, Parr WCH, Stynder DD, Wroe S 2012 Finite element analysis of ursid cranial mechanics and the prediction of feeding behaviour in the extinct giant *Agriotherium africanum*. J. Zool. 286, 163–170. (10.1111/j.1469-7998.2011.00862.x)

[21] Snively E, Russell A 2002 The Tyrannosaurid metatarsus: bone strain and inferred ligament function. Senck. leth. 82, 35–42. (10.1007/bf03043771)

[22] Manning PL, Margetts L, Johnson MR, Withers PJ, Sellers WI, Falkingham PL, Mummery PM, Barrett PM, Raymont DR 2009 Biomechanics of dromaeosaurid dinosaur claws: application of X-ray microtomography, nanoindentation, and finite element analysis. Anat. Rec. 292, 1397–1405. (10.1002/ar.20986) 19711472

[23] Falkingham PL, Bates KT, Margetts L, Manning PL 2011 Simulating sauropod manus-only trackway formation using finite-element analysis. Biol. Lett. 7, 142–145. (10.1098/rsbl.2010.0403) 20591856 PMC3030862

[24] Bishop PJ, Hocknull SA, Clemente CJ, Hutchinson JR, Barrett RS, Lloyd DG 2018a Cancellous bone and theropod dinosaur locomotion. Part II—a new approach to inferring posture and locomotor biomechanics in extinct tetrapod vertebrates. PeerJ 6, e5779 (10.7717/peerj.5779) 30402348 PMC6215447

[25] Bishop PJ, Hocknull SA, Clemente CJ, Hutchinson JR, Farke AA, Barrett RS, Lloyd DG 2018 Cancellous bone and theropod dinosaur locomotion. Part III—inferring posture and locomotor biomechanics in extinct theropods, and its evolution on the line to birds. PeerJ 6, e5777 (10.7717/peerj.5777) 30402346 PMC6215443

[26] Ross CF, Berthaume MA, Dechow PC, Iriarte-Diaz J, Porro LB, Richmond BG, Spencer M, Strait D 2011 In vivo bone strain and finite-element modeling of the craniofacial haft in catarrhine primates. J. Anat. 218, 112–141. (10.1111/j.1469-7580.2010.01322.x) 21105871 PMC3039785

[27] Metzger KA, Daniel WJT, Ross CF 2005 Comparison of beam theory and finite-element analysis with in vivo bone strain data from the alligator cranium. Anat. Rec. Part A 283A, 331–348. (10.1002/ar.a.20167) 15747347

[28] Porro LB, Metzger KA, Iriarte-Diaz J, Ross CF 2013 *In vivo* bone strain and finite element modeling of the mandible of *Alligator mississippiensis*. J. Anat. 223, 195–227. (10.1111/joa.12080) 23855772 PMC3972043

[29] Marinescu R, Daegling DJ, Rapoff AJ 2005 Finite-element modeling of the anthropoid mandible: the effects of altered boundary conditions. Anat. Rec. Part A 283A, 300–309. (10.1002/ar.a.20166) 15747352

[30] Panagiotopoulou O, Iriarte-Diaz J, Wilshin S, Dechow PC, Taylor AB, Abraha HM, Aljunid SF, Ross CF 2017 In vivo bone strain and finite element modeling of a rhesus macaque mandible during mastication. Zoology 124, 13–29. (10.1016/j.zool.2017.08.010) 29037463 PMC5792078

[31] Strait DS, Wang Q, Dechow PC, Ross CF, Richmond BG, Spencer MA, Patel BA 2005 Modeling elastic properties in finite-element analysis: how much precision is needed to produce an accurate model? Anat. Rec. Part A 283A, 275–287. (10.1002/ar.a.20172) 15747346

[32] Ross CF, Patel BA, Slice DE, Strait DS, Dechow PC, Richmond BG, Spencer MA 2005 Modeling masticatory muscle force in finite element analysis: sensitivity analysis using principal coordinates analysis. Anat. Rec. Part A 283A, 288–299. (10.1002/ar.a.20170) 15747351

[33] Kupczik K, Dobson CA, Fagan MJ, Crompton RH, Oxnard CE, O'Higgins P 2005 Assessing mechanical function of the zygomatic region in macaques: validation and sensitivity testing of finite element models. J. Anat. 210, 41–53. (10.1111/j.1469-7580.2006.00662.x) PMC2100262 17229282

[34] Tseng ZJ, Mcnitt-Gray JL, Flashner H, Wang X, Enciso R 2011 Model sensitivity and use of the comparative finite element method in mammalian jaw mechanics: mandible performance in the gray wolf. PLoS ONE 6, e19171 (10.1371/journal.pone.0019171) 21559475 PMC3084775

[35] Bright JA, Rayfield EJ 2011 The response of cranial biomechanical finite element models to variations in mesh density. Anat Rec. 294, 610–620. (10.1002/ar.21358) 21370496

[36] Marce-Nogue J, Fortuny J, Gil L, Sánchez M 2015 Improving mesh generation in finite element analysis for functional morphology approaches. Span. J. Palaeontol. 30, 117–132.

[37] Marce-Nogue J, de Esteban-Trivigno S, Escrig C, Gil L. 2016 Accounting for differences in element size and homogeneity when comparing finite element models: armadillos as a case study. Palaeontol. Electron. 19, 1–22. (10.26879/609)

[38] Foffa D, Cuff AR, Sassoon J, Rayfield EJ, Mavrogordato MN, Benton MJ 2014 Functional anatomy and feeding biomechanics of a giant Upper Jurassic pliosaur (Reptilia: Sauropterygia) from Weymouth Bay, Dorset, UK. J. Anat. 225, 209–219. (10.1111/joa.12200) 24925465 PMC4111928

[39] Lautenschlager S, Gill P, Luo ZX, Fagan MJ, Rayfield EJ 2016 Morphological evolution of the mammalian jaw adductor complex. Biol. Rev. 92, 1910–1940. (10.1111/brv.12314) 27878942 PMC6849872

[40] Lautenschlager S, Gill PG, Luo ZX, Fagan MJ, Rayfield EJ 2018 The role of miniaturization in the evolution of the mammalian jaw and middle ear. Nature 561, 533–537. (10.1038/s41586-018-0521-4) 30224748

[41] Bright JA 2014 A review of paleontological finite element models and their validity. J. Paleontol. 88, 760–769. (10.1666/13-090)

[42] Sutton MD, Rahman IA, Garwood RJ 2013 Surface-based methods. In Techniques for virtual palaeontology (eds Sutton MD, Rahman IA, Garwood RJ), pp. 115–129. John Wiley & Sons Ltd.

[43] Neenan JM, Ruta M, Clack JA, Rayfield EJ 2014 Feeding biomechanics in *Acanthostega* and across the fish–tetrapod transition. Proc. R. Soc. B 281, 20132689 (10.1098/rspb.2013.2689) PMC3953833 24573844

[44] Fortuny J, Marcé-Nogué J, De Esteban-Trivigno S, Gil L, Galobart À 2011 Temnospondyli bite club: ecomorphological patterns of the most diverse group of early tetrapods. J. Evol. Biol. 24, 2040–2054. (10.1111/j.1420-9101.2011.02338.x) 21707813

[45] Fortuny J, Marce-Nogue J, Gil L, Galobart A 2012 Skull mechanics and the evolutionary patterns of the otic notch closure in capitosaurs (Amphibia: Temnospondyli). Anat. Rec. 295, 1134–1146. (10.1002/ar.22486) 22573567

[46] Rayfield EJ 2005 Using finite-element analysis to investigate suture morphology: a case study using large carnivorous dinosaurs. Anat. Rec. Part A 283A, 349–365. (10.1002/ar.a.20168) 15751029

[47] Rayfield EJ 2005 Aspects of comparative cranial mechanics in the theropod dinosaurs *Coelophysis*, *Allosaurus* and *Tyrannosaurus*. Zool. J. Linn. Soc. Lond. 144, 309–316. (10.1111/j.1096-3642.2005.00176.x)

[48] Maiorino L, Farke AA, Kotsakis T, Teresi L, Piras P 2015 Variation in the shape and mechanical performance of the lower jaws in ceratopsid dinosaurs (Ornithischia, Ceratopsia). J. Anat. 227, 631–646. (10.1111/joa.12374) 26467240 PMC4609198

[49] Serrano-Fochs S, De Esteban-Trivigno S, Marce-Nogue J, Fortuny J, Farina RA. 2015 Finite element analysis of the cingulata jaw: an ecomorphological approach to armadillo's diets. PLoS ONE 10, e0120653 (10.1371/journal.pone.0120653) 25919313 PMC4412537

[50] Marcé-Nogué J, Püschel TA, Kaiser TM 2017 A biomechanical approach to understand the ecomorphological relationship between primate mandibles and diet. Sci. Rep. UK 7, 8364 (10.1038/s41598-017-08161-0) PMC5567063 28827696

[51] Fletcher TM, Janis CM, Rayfield EJ 2010 Finite element analysis of ungulate jaws: can mode of digestive physiology be determined? Palaeontol. Electron. 13, 1–15.

[52] Schneider CA, Rasband WS, Eliceiri KW 2012 NIH Image to ImageJ: 25 years of image analysis. Nat. Methods 9, 671 (10.1038/nmeth.2089) 22930834 PMC5554542

[53] Rahman IA, Lautenschlager S 2016 Applications of the three-dimensional box modelling to paleontological functional analysis. Paleontol. Soc. Pap. 22, 119–132. (10.1017/scs.2017.11)

