# Supplementary material for: The use of extruded finite-element models as a novel alternative to tomography-based models: a case study using early mammal jaws
Source: J R Soc Interface. 2019 Dec 11;16(161):20190674. doi: 10.1098/rsif.2019.0674 (PMC6936041; doi:10.1098/rsif.2019.0674)

Journal of the Royal Society Interface

SUPPLEMENTARY INFORMATION

The use of extruded finite element models as a novel alternative to tomography-based models: a case study using early mammal jaws

Nuria Melisa Morales-García, Thomas D. Burgess, Jennifer J. Hill, Pamela G. Gill and Emily J. Rayfield.

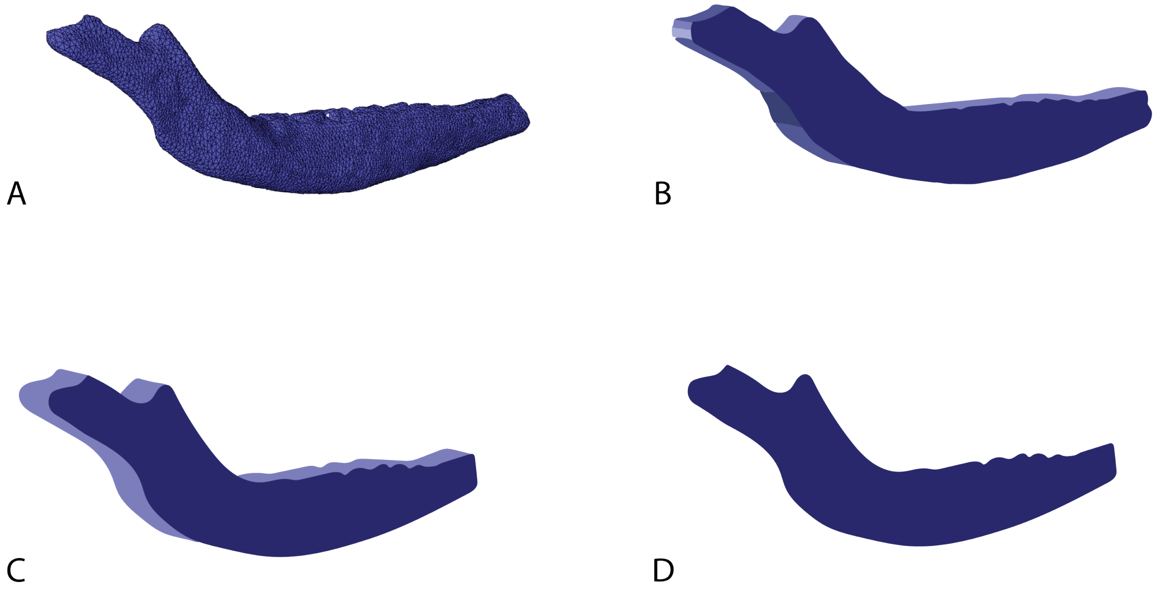

Figure 1. FE models analysed in this paper, using the example of *Kuehneotherium*: (A) CT scan-based 3D model, (B) Enhanced extruded model, (C) extruded model and (D) 2D planar model

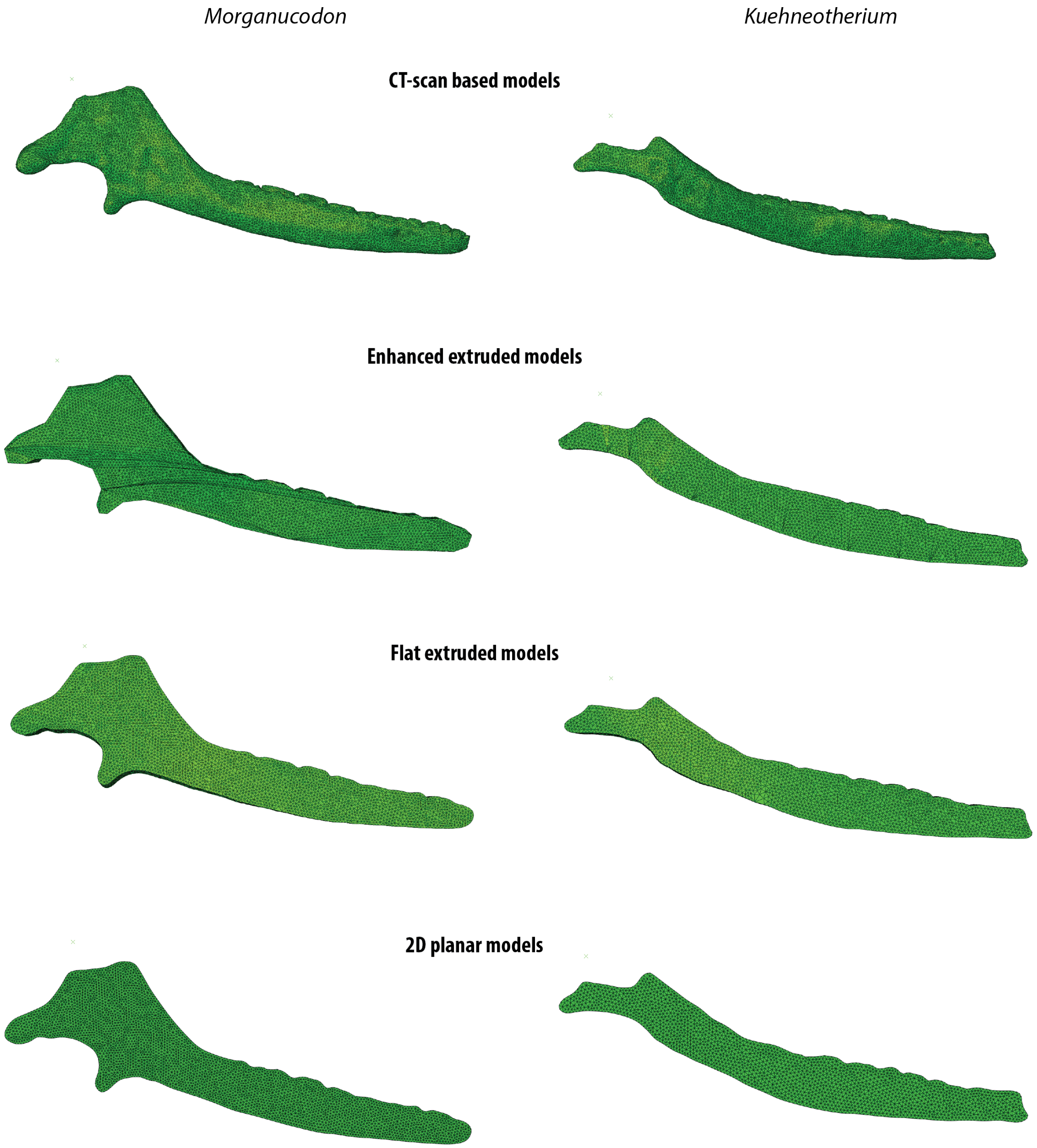

Figure 2. Deformation patterns in all FE models evaluated in this study for both *Morganucodon* (left) and *Kuehneotherium* (right).

Table 1. Summary of elements in the mesh of *Morganucodon* and *Kuehneotherium.*

Note that the 3D, enhanced extruded and extruded models use linear four-noded tetrahedral (C3D4) elements, while the planar 2D model uses 3 node linear triangular (CPE3) elements.

	Morganucodon	*Kuehneotherium*
3D model	115213	68555
Enhanced 2D extruded model	111770	68074
Flat 2D extruded model	115420	68239
Planar 2D model	7192	3550

Table 2. Comparative results of biomechanical analyses- *Morganucodon* and *Kuehneotherium* under the two different FE models: **A-**Two material properties: bone and dentine (Young's modulus=25 GPa, Poisson's ratio=0.3), **B-**One material property: bone. Green: more than 75% similarity with values obtained from 3D model; Yellow: between 50-74%; Red: less than 50% similarity.

	Morganucodon		***Kuehneotherium***	
	A	B	A	B
**Von Mises stress (MPa)**				
• Mean	3.99	3.96	4.21	4.18
• Median	3.00	2.93	2.08	2.04
• Max	53.8	53.41	82.7	82.84
**Max. principal strain (microstrain)**				
• Mean	142	142	156	158
• Median	102	101.5	73.5	75.1
• Max	3,100	3,084	4,920	4,930
**Reaction forces (N)**				
• Jaw joint	2.38	2.38	3.12	3.12
• Bite	2.00	2.00	1.14	1.14

Table 3. Sensitivity analyses method

The sensitivity analyses were carried out by moving the muscle loads 1%, 5% and 10% of the total length of the jaw in the x, y and z axis as follows:

Analysis number	Change in x axis	Change in y axis	Change in z axis
1	0	Negative	0
2	0	Positive	0
3	Positive	0	0
4	Negative	0	0
5	Positive	Negative	0
6	Negative	Negative	0
7	Negative	Positive	0
8	Positive	Positive	0
9	0	Negative	Negative
10	0	Negative	Positive
11	0	Positive	Positive
12	0	Positive	Negative
13	Positive	0	Negative
14	Positive	0	Positive
15	Negative	0	Positive
16	Negative	0	Negative
17	0	0	Negative
18	0	0	Positive
19	Positive	Positive	Positive
20	Negative	Negative	Negative
21	Positive	Positive	Negative
22	Negative	Negative	Positive
23	Positive	Negative	Positive
24	Negative	Positive	Negative
25	Negative	Positive	Positive
26	Positive	Negative	Negative

x axis: ***positive***=towards the anterior end of the jaw, ***negative***= towards the posterior end of the jaw; **y axis:** ***positive***= towards the dorsal end of the jaw, ***negative***= towards the ventral end of the jaw; **z axis:** ***positive***= towards the labial end of the jaw, ***negative***= towards the lingual end of the jaw.

Table 4. Sensitivity analyses

Morganucodon- Moving all muscles 1%

Raw values (Microstrain; Von Mises stress in MPa)

	Strain			Stress			Validity
	**Mean**	**Median**	**Max**	**Mean**	**Median**	**Max**	
**1**	137.2	90.9	2722.7	3.6	2.8	55.5	Valid
**2**	135.5	90.9	2719.1	3.6	2.8	55.3	Invalid
**3**	136.8	91.5	2665.0	3.6	2.8	55.2	Valid
**4**	135.8	90.4	2610.1	3.6	2.7	55.6	Valid
**5**	136.0	91.5	2797.9	3.6	2.8	55.2	Invalid
**6**	135.0	90.5	2641.3	3.6	2.8	55.5	Invalid
**7**	136.7	90.2	2735.8	3.6	2.7	55.7	Valid
**8**	137.7	91.4	2718.5	3.6	2.8	55.3	Valid
**9**	142.9	94.5	2805.2	3.7	2.9	55.7	Invalid
**10**	131.6	92.0	2637.4	3.5	2.8	54.9	Valid
**11**	132.3	90.5	2641.1	3.5	2.8	55.1	Valid
**12**	145.4	95.6	2942.7	3.8	2.9	55.9	Invalid
**13**	144.7	95.7	2829.8	3.8	2.9	55.7	Invalid
**14**	132.2	91.5	2583.3	3.5	2.8	54.8	Valid
**15**	131.6	90.9	2581.5	3.5	2.8	55.2	Valid
**16**	143.6	94.4	2787.5	3.7	2.8	56.0	Invalid
**17**	144.1	95.1	2805.0	3.8	2.9	55.8	Invalid
**18**	131.9	91.2	2529.4	3.5	2.8	55.0	Valid
**19**	132.7	90.8	2600.8	3.5	2.8	54.9	Valid
**20**	142.4	93.9	2725.4	3.7	2.8	55.9	Invalid
**21**	146.0	96.2	2968.2	3.8	2.9	55.8	Invalid
**22**	131.3	91.8	2561.9	3.5	2.8	55.1	Valid
**23**	131.9	92.1	2714.0	3.5	2.8	54.7	Valid
**24**	144.9	95.1	2924.1	3.8	2.8	56.1	Invalid
**25**	132.0	90.1	2689.6	3.5	2.8	55.3	Valid
**26**	143.5	95.1	2885.8	3.7	2.9	55.6	Invalid
**Original**	136.3	90.9	2590.0	3.6	2.8	55.4	
**Min**	131.3	90.1	2529.4	3.5	2.7	54.7	
**Max**	146.0	96.2	2968.2	3.8	2.9	56.1	
**STD**	5.3	2.0	121.0	0.1	0.04	0.4	

Table 4a. Graphic representation of how similar they are to the original values obtained from the enhanced extruded FE models (percentually)

	Strain			Stress			Validity
	Mean	Median	Max	Mean	Median	Max	
1	100.67	100.04	105.13	100.45	99.56	100.20	Valid
2	99.41	99.96	104.98	99.63	100.30	99.85	Invalid
3	100.38	100.63	102.89	100.33	100.54	99.73	Valid
4	99.67	99.51	100.78	99.72	99.12	100.33	Valid
5	99.79	100.69	108.03	99.96	100.97	99.55	Invalid
6	99.09	99.54	101.98	99.35	99.54	100.16	Invalid
7	100.34	99.27	105.63	100.16	98.90	100.50	Valid
8	101.05	100.56	104.96	100.79	100.38	99.90	Valid
9	104.87	104.03	108.31	104.22	103.07	100.63	Invalid
10	96.57	101.25	101.83	97.46	101.75	99.08	Valid
11	97.09	99.52	101.97	97.59	100.02	99.43	Valid
12	106.70	105.23	113.62	105.59	103.29	100.98	Invalid
13	106.21	105.30	109.26	105.29	103.84	100.50	Invalid
14	97.04	100.64	99.74	97.70	101.22	98.95	Valid
15	96.59	100.04	99.67	97.31	100.24	99.56	Valid
16	105.35	103.84	107.63	104.51	102.45	101.10	Invalid
17	105.75	104.60	108.30	104.87	103.07	100.80	Invalid
18	96.79	100.36	97.66	97.48	100.93	99.25	Valid
19	97.36	99.92	100.42	97.81	100.53	99.13	Valid
20	104.47	103.28	105.23	103.86	102.44	100.93	Invalid
21	107.15	105.82	114.60	106.00	103.86	100.68	Invalid
22	96.37	100.96	98.91	97.29	101.36	99.38	Valid
23	96.82	101.36	104.79	97.68	102.06	98.78	Valid
24	106.31	104.58	112.90	105.23	102.74	101.28	Invalid
25	96.88	99.08	103.84	97.41	99.52	99.73	Valid
26	105.33	104.61	111.42	104.63	103.73	100.33	Invalid

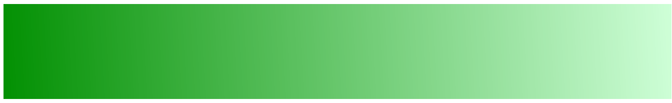

100% Similarity 0%

Table 5. Sensitivity analyses

Morganucodon- Moving all muscles 5%

Raw values (Microstrain; Von Mises stress in MPa)

	Strain			Stress			Validity
	**Mean**	**Median**	**Max**	**Mean**	**Median**	**Max**	
**1**	141.9	92.1	3269.1	3.7	2.8	58.9	Valid
**2**	133.4	91.9	3255.4	3.5	2.9	54.9	Invalid
**3**	139.6	93.0	2985.1	3.7	2.8	54.6	Valid
**4**	134.8	87.2	2760.7	3.5	2.7	56.3	Invalid
**5**	136.5	92.7	3668.4	3.6	2.9	58.8	Invalid
**6**	132.1	88.7	2858.1	3.5	2.7	55.8	Invalid
**7**	140.5	89.4	3366.7	3.7	2.7	56.7	Valid
**8**	145.1	94.9	3350.9	3.8	2.8	66.0	Valid
**9**	185.2	122.8	3741.3	4.7	3.6	77.6	Invalid
**10**	159.8	118.5	3142.6	4.2	3.4	59.3	Invalid
**11**	145.0	104.0	3706.4	3.8	3.0	57.7	Invalid
**12**	205.2	136.5	5068.0	5.2	3.9	110.0	Invalid
**13**	199.2	132.4	4738.0	5.1	3.8	104.0	Invalid
**14**	151.1	109.6	2829.3	4.0	3.2	52.4	Invalid
**15**	152.6	108.9	3947.9	4.0	3.1	69.1	Invalid
**16**	191.5	127.3	4028.7	4.9	3.7	83.0	Invalid
**17**	194.5	129.3	4363.6	4.9	3.7	93.0	Invalid
**18**	150.8	109.2	3376.9	4.0	3.1	57.2	Invalid
**19**	145.5	105.2	3186.8	3.8	3.0	52.9	Invalid
**20**	181.9	120.4	3339.0	4.6	3.5	66.2	Invalid
**21**	209.6	139.5	5426.1	5.3	4.0	120.0	Invalid
**22**	161.6	118.5	3712.8	4.2	3.4	71.4	Invalid
**23**	160.0	118.6	3222.6	4.2	3.5	52.0	Invalid
**24**	202.5	134.3	4749.9	5.1	3.8	101.1	Invalid
**25**	146.6	102.8	4260.1	3.9	2.9	69.7	Invalid
**26**	190.1	126.2	4175.8	4.9	3.7	89.7	Invalid
**Original**	136.3	90.9	2590.0	3.6	2.8	55.4	
**Min**	132.1	87.2	2760.7	3.5	2.7	52.0	
**Max**	209.6	139.5	5426.1	5.3	4.0	120.0	
**STD**	25.8	16.8	712.5	0.6	0.42	20.0	

Table 5a. Graphic representation of how similar they are to the original values obtained from the enhanced extruded FE models (percentually)

	Strain			Stress			Validity
	**Mean**	**Median**	**Max**	**Mean**	**Median**	**Max**	
**1**	104.10	101.35	126.22	103.02	99.60	106.35	Valid
**2**	97.86	101.15	125.69	98.97	102.99	99.16	Invalid
**3**	102.42	102.37	115.25	102.15	102.71	98.54	Valid
**4**	98.90	95.91	106.59	99.03	95.97	101.55	Invalid
**5**	100.14	101.98	141.64	100.91	103.19	106.12	Invalid
**6**	96.91	97.55	110.35	98.11	98.94	100.68	Invalid
**7**	103.08	98.38	129.99	102.10	96.71	102.42	Valid
**8**	106.50	104.38	129.38	105.23	102.75	119.18	Valid
**9**	135.89	135.09	144.45	132.06	130.00	140.02	Invalid
**10**	117.30	130.38	121.34	117.43	122.55	106.99	Invalid
**11**	106.40	114.47	143.10	106.74	107.84	104.18	Invalid
**12**	150.61	150.21	195.67	144.79	139.97	198.62	Invalid
**13**	146.19	145.62	182.93	141.25	137.82	187.64	Invalid
**14**	110.85	120.62	109.24	111.52	114.83	94.67	Invalid
**15**	111.96	119.86	152.43	112.00	111.89	124.64	Invalid
**16**	140.54	140.01	155.55	135.78	131.79	149.79	Invalid
**17**	142.76	142.28	168.48	137.92	134.95	167.89	Invalid
**18**	110.66	120.10	130.38	111.12	113.23	103.28	Invalid
**19**	106.75	115.78	123.04	107.07	109.01	95.55	Invalid
**20**	133.47	132.44	128.92	129.78	127.20	119.45	Invalid
**21**	153.83	153.54	209.50	147.95	142.90	216.53	Invalid
**22**	118.62	130.43	143.35	118.18	121.06	128.82	Invalid
**23**	117.39	130.49	124.43	117.90	124.55	93.80	Invalid
**24**	148.61	147.72	183.39	142.84	137.77	182.44	Invalid
**25**	107.55	113.13	164.48	107.68	106.37	125.90	Invalid
**26**	139.53	138.82	161.23	135.53	133.09	161.91	Invalid

100% Similarity 0%

Table 6. Sensitivity analyses

Morganucodon- Moving all muscles 10%

Raw values (Microstrain; Von Mises stress in MPa)

	Strain			Stress			Validity
	**Mean**	**Median**	**Max**	**Mean**	**Median**	**Max**	
**1**	149.9	96.5	3999.5	3.9	2.9	78.8	Valid
**2**	134.0	95.0	3938.1	3.6	3.0	62.7	Invalid
**3**	144.8	95.6	3399.8	3.8	2.9	58.9	Valid
**4**	135.0	82.7	3108.3	3.5	2.5	57.1	Invalid
**5**	141.3	90.4	4788.6	3.7	2.8	77.9	Invalid
**6**	134.0	87.6	3130.0	3.6	2.7	56.2	Invalid
**7**	149.5	93.2	4254.2	3.8	2.7	73.2	Valid
**8**	157.9	102.3	4305.9	4.1	3.0	92.9	Valid
**9**	254.7	169.3	5427.7	6.4	4.7	121.3	Invalid
**10**	246.8	182.6	4815.6	6.3	5.0	114.3	Invalid
**11**	201.3	148.5	5653.2	5.2	4.1	103.2	Invalid
**12**	300.9	197.6	8086.2	7.5	5.3	181.8	Invalid
**13**	287.4	188.7	7566.3	7.2	5.1	172.4	Invalid
**14**	218.8	155.5	3947.4	5.6	4.4	79.3	Invalid
**15**	228.2	166.4	6345.2	5.8	4.5	130.2	Invalid
**16**	269.8	175.8	5918.8	6.7	4.9	127.7	Invalid
**17**	275.9	183.2	6718.8	6.9	5.0	149.1	Invalid
**18**	220.4	162.6	5138.7	5.6	4.5	104.6	Invalid
**19**	199.4	143.3	4469.2	5.1	4.0	77.1	Invalid
**20**	247.5	162.7	4584.3	6.2	4.6	96.4	Invalid
**21**	311.2	203.1	8894.3	7.7	5.5	202.6	Invalid
**22**	253.3	186.0	5986.8	6.4	5.0	139.3	Invalid
**23**	246.2	177.5	4214.1	6.3	5.0	89.8	Invalid
**24**	295.9	190.7	7336.5	7.4	5.2	163.1	Invalid
**25**	209.6	150.2	6856.3	5.4	4.2	129.4	Invalid
**26**	267.4	175.0	6307.3	6.7	4.8	147.3	Invalid
**Original**	136.3	90.9	2590.0	3.6	2.8	55.4	
**Min**	134.0	82.7	3108.3	3.5	2.5	56.2	
**Max**	311.2	203.1	8894.3	7.7	5.5	202.6	
**STD**	58.7	40.5	1560.9	1.4	1.00	41.1	

Table 6a. Graphic representation of how similar they are to the original values obtained from the enhanced extruded FE models (percentually)

	Strain			Stress			Validity
	Mean	Median	Max	Mean	Median	Max	
1	110.01	106.18	154.42	107.86	102.95	142.28	Valid
2	98.35	104.54	152.05	100.20	106.74	113.25	Invalid
3	106.25	105.22	131.27	105.67	104.57	106.26	Valid
4	99.11	90.97	120.01	99.16	90.64	103.10	Invalid
5	103.68	99.43	184.89	104.68	100.16	140.60	Invalid
6	98.31	96.36	120.85	99.69	96.53	101.38	Invalid
7	109.70	102.57	164.25	107.49	98.97	132.06	Valid
8	115.90	112.56	166.25	113.50	108.96	167.72	Valid
9	186.89	186.27	209.56	178.71	170.19	218.97	Invalid
10	181.13	200.88	185.93	175.46	181.14	206.28	Invalid
11	147.70	163.43	218.27	144.41	148.58	186.29	Invalid
12	220.82	217.35	312.21	209.10	192.91	328.13	Invalid
13	210.93	207.66	292.14	200.65	185.75	311.20	Invalid
14	160.56	171.06	152.41	157.33	159.33	143.15	Invalid
15	167.48	183.05	244.99	162.00	162.34	235.02	Invalid
16	197.98	193.44	228.52	188.29	175.22	230.51	Invalid
17	202.45	201.58	259.41	192.50	181.05	269.16	Invalid
18	161.73	178.90	198.40	157.41	161.10	188.84	Invalid
19	146.37	157.69	172.55	143.77	144.24	139.24	Invalid
20	181.62	178.99	177.00	173.79	165.25	174.03	Invalid
21	228.39	223.50	343.41	216.40	197.97	365.67	Invalid
22	185.90	204.59	231.15	178.95	180.39	251.46	Invalid
23	180.68	195.27	162.71	176.10	181.44	162.09	Invalid
24	217.19	209.85	283.26	205.70	187.79	294.45	Invalid
25	153.81	165.29	264.72	149.56	150.50	233.64	Invalid
26	196.25	192.52	243.52	187.63	174.37	265.88	Invalid

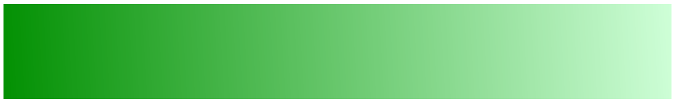

100% Similarity 0%

Table 7. Sensitivity analyses

Kuehneotherium- Moving all muscles 1%

Raw values (Microstrain; Von Mises stress in MPa)

	Strain			Stress			Validity
	**Mean**	**Median**	**Max**	**Mean**	**Median**	**Max**	
**1**	133.9	55.2	5405.7	3.7	1.7	85.0	Valid
**2**	131.3	56.3	5387.5	3.7	1.6	84.7	Valid
**3**	133.8	57.3	5272.0	3.7	1.7	82.9	Valid
**4**	131.3	54.2	5521.2	3.7	1.6	86.8	Valid
**5**	132.5	57.6	5263.0	3.7	1.7	82.7	Valid
**6**	130.2	54.6	5512.1	3.6	1.6	86.6	Valid
**7**	132.7	53.7	5530.4	3.7	1.6	86.9	Valid
**8**	135.3	56.6	5281.1	3.8	1.7	83.0	Valid
**9**	132.0	48.9	5701.7	3.7	1.5	89.7	Invalid
**10**	136.7	64.2	5077.1	3.8	1.8	79.8	Valid
**11**	137.7	61.5	5094.9	3.8	1.7	80.1	Valid
**12**	136.4	49.8	5720.2	3.8	1.6	90.0	Invalid
**13**	135.7	50.8	5586.1	3.7	1.6	87.8	Invalid
**14**	138.0	63.8	4961.7	3.8	1.8	78.0	Valid
**15**	136.4	61.8	5210.2	3.8	1.7	81.9	Valid
**16**	132.7	47.6	5835.8	3.7	1.5	91.8	Invalid
**17**	134.1	49.3	5710.9	3.7	1.5	89.8	Invalid
**18**	137.1	62.8	5086.0	3.8	1.7	79.9	Valid
**19**	138.7	62.9	4970.6	3.8	1.8	78.1	Valid
**20**	130.7	47.2	5826.5	3.6	1.5	91.6	Invalid
**21**	138.0	51.5	5595.3	3.8	1.6	88.0	Invalid
**22**	136.1	63.4	5201.3	3.8	1.7	81.7	Valid
**23**	137.4	65.0	4952.9	3.8	1.8	77.8	Valid
**24**	134.9	48.1	5845.0	3.7	1.5	91.9	Invalid
**25**	136.9	60.5	5219.2	3.8	1.7	82.0	Valid
**26**	133.6	50.6	5576.9	3.7	1.6	87.7	Invalid
**Original**	132.5	55.8	5396.6	3.7	1.6	84.8	
**Min**	130.2	47.2	4952.9	3.6	1.5	77.8	
**Max**	138.7	65.0	5845.0	3.8	1.8	91.9	
**STD**	2.5	5.9	285.5	0.1	0.10	4.5	

Table 7a. Graphic representation of how similar they are to the original values obtained from the enhanced extruded FE models (percentually)

	Strain			Stress			Validity
	Mean	Median	Max	Mean	Median	Max	
**1**	101.08	98.94	100.17	100.91	100.67	100.17	Valid
**2**	99.06	100.80	99.83	99.22	99.82	99.83	Valid
**3**	100.99	102.59	97.69	100.93	103.30	97.69	Valid
**4**	99.11	97.02	102.31	99.16	96.81	102.31	Valid
**5**	99.99	103.19	97.52	100.11	103.36	97.51	Valid
**6**	98.23	97.82	102.14	98.42	96.31	102.14	Valid
**7**	100.14	96.18	102.48	100.02	97.18	102.49	Valid
**8**	102.12	101.38	97.86	101.89	103.83	97.86	Valid
**9**	99.66	87.53	105.65	99.35	92.80	105.71	Invalid
**10**	103.15	115.03	94.08	103.08	107.30	94.04	Valid
**11**	103.95	110.23	94.41	103.70	105.01	94.38	Valid
**12**	102.94	89.19	106.00	102.17	94.14	106.07	Invalid
**13**	102.40	91.03	103.51	101.79	95.92	103.57	Invalid
**14**	104.14	114.36	91.94	103.98	108.17	91.91	Valid
**15**	102.93	110.69	96.55	102.76	103.55	96.52	Valid
**16**	100.14	85.24	108.14	99.68	90.03	108.21	Invalid
**17**	101.22	88.28	105.82	100.70	92.96	105.89	Invalid
**18**	103.47	112.43	94.24	103.32	105.67	94.21	Valid
**19**	104.70	112.60	92.11	104.42	107.81	92.08	Valid
**20**	98.61	84.59	107.97	98.36	89.54	108.03	Invalid
**21**	104.15	92.17	103.68	103.29	97.17	103.75	Invalid
**22**	102.70	113.55	96.38	102.58	105.21	96.35	Valid
**23**	103.73	116.41	91.78	103.67	109.37	91.74	Valid
**24**	101.82	86.11	108.31	101.14	91.11	108.39	Invalid
**25**	103.32	108.30	96.71	103.07	102.25	96.69	Valid
**26**	100.80	90.65	103.34	100.42	96.07	103.39	Invalid

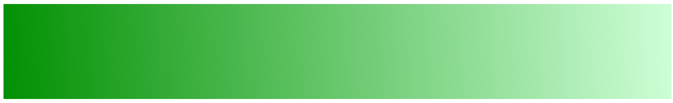

Table 8. Sensitivity analyses

Kuehneotherium- Moving all muscles 5%

Raw values (Microstrain; Von Mises stress in MPa)

	Strain			Stress			Validity
	**Mean**	**Median**	**Max**	**Mean**	**Median**	**Max**	
**1**	141.4	52.1	5442.2	3.9	1.6	90.6	Valid
**2**	128.4	57.9	5351.3	3.6	1.6	84.1	Invalid
**3**	140.7	62.5	4774.1	3.9	1.9	75.0	Valid
**4**	127.8	47.3	6020.0	3.6	1.4	94.6	Valid
**5**	134.8	63.7	4729.1	3.8	1.9	74.3	Invalid
**6**	125.9	52.3	5974.3	3.5	1.4	93.9	Invalid
**7**	135.0	43.9	6065.8	3.7	1.4	99.7	Valid
**8**	150.9	60.0	4819.2	4.1	1.9	82.5	Valid
**9**	170.1	101.7	7033.5	4.5	2.9	109.6	Invalid
**10**	203.9	146.8	4236.0	5.4	4.0	76.2	Invalid
**11**	191.8	123.9	4614.3	5.1	3.4	85.4	Invalid
**12**	205.2	128.7	7158.7	5.4	3.7	115.2	Invalid
**13**	196.1	117.9	6460.0	5.2	3.4	100.6	Invalid
**14**	196.5	132.1	3834.1	5.2	3.7	65.4	Invalid
**15**	199.7	137.9	4529.2	5.3	3.7	81.8	Invalid
**16**	180.4	110.8	7731.7	4.8	3.1	120.3	Invalid
**17**	186.8	115.8	7095.8	4.9	3.3	110.4	Invalid
**18**	196.5	135.7	4124.8	5.2	3.7	71.4	Invalid
**19**	193.4	121.9	4011.9	5.1	3.4	71.0	Invalid
**20**	164.1	95.5	7669.5	4.4	2.7	119.4	Invalid
**21**	214.9	131.7	6799.7	5.6	3.8	112.2	Invalid
**22**	208.2	151.2	4528.5	5.5	4.0	82.1	Invalid
**23**	202.6	141.5	3987.9	5.4	3.9	70.2	Invalid
**24**	198.4	125.6	7794.5	5.2	3.6	121.1	Invalid
**25**	193.5	124.0	5259.0	5.1	3.4	100.4	Invalid
**26**	179.3	105.7	6397.6	4.8	3.0	99.7	Invalid
**Original**	132.5	55.8	5396.6	3.7	1.6	84.8	
**Min**	125.9	43.9	3834.1	3.5	1.4	65.4	
**Max**	214.9	151.2	7794.5	5.6	4.0	121.1	
**STD**	29.6	35.5	1288.1	0.7	0.93	17.4	

Table 8a. Graphic representation of how similar they are to the original values obtained from the enhanced extruded FE models (percentually)

	Strain			Stress			Validity
	Mean	Median	Max	Mean	Median	Max	
**1**	106.71	93.26	100.84	105.77	99.16	106.78	Valid
**2**	96.91	103.80	99.16	97.57	97.91	99.13	Invalid
**3**	106.21	112.00	88.46	105.63	116.68	88.44	Valid
**4**	96.42	84.69	111.55	96.55	82.17	111.58	Valid
**5**	101.76	114.03	87.63	102.11	112.88	87.58	Invalid
**6**	95.00	93.65	110.70	95.48	86.97	110.70	Invalid
**7**	101.91	78.60	112.40	101.25	83.03	117.52	Valid
**8**	113.90	107.42	89.30	112.14	115.36	97.22	Valid
**9**	128.40	182.12	130.33	123.15	175.16	129.20	Invalid
**10**	153.88	263.05	78.49	145.67	243.04	89.78	Invalid
**11**	144.76	222.01	85.50	138.12	206.24	100.72	Invalid
**12**	154.90	230.65	132.65	146.31	224.34	135.78	Invalid
**13**	148.00	211.25	119.70	140.40	207.04	118.56	Invalid
**14**	148.32	236.65	71.05	141.45	221.52	77.11	Invalid
**15**	150.72	246.97	83.93	142.65	225.84	96.41	Invalid
**16**	136.15	198.58	143.27	129.62	189.43	141.80	Invalid
**17**	140.95	207.51	131.49	134.03	199.52	130.18	Invalid
**18**	148.32	243.13	76.43	140.99	224.77	84.17	Invalid
**19**	145.96	218.38	74.34	139.61	204.71	83.69	Invalid
**20**	123.87	171.13	142.12	118.88	162.73	140.83	Invalid
**21**	162.17	235.89	126.00	152.77	231.11	132.31	Invalid
**22**	157.14	270.78	83.91	148.14	245.60	96.85	Invalid
**23**	152.91	253.51	73.90	145.25	237.39	82.72	Invalid
**24**	149.74	224.94	144.43	141.69	215.98	142.77	Invalid
**25**	146.01	222.13	97.45	138.72	205.17	118.33	Invalid
**26**	135.31	189.27	118.55	129.42	183.89	117.59	Invalid

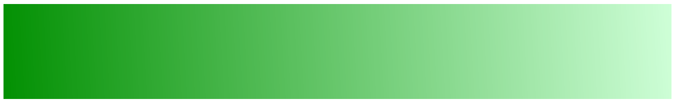

100% Similarity 0%

Table 9. Sensitivity analyses

Kuehneotherium- Moving all muscles 10%

Raw values (Microstrain; Von Mises stress in MPa)

	Strain			Stress			Validity
	**Mean**	**Median**	**Max**	**Mean**	**Median**	**Max**	
**1**	154.4	54.9	6687.8	4.2	1.7	124.3	Valid
**2**	130.6	66.2	5306.1	3.7	1.8	83.4	Invalid
**3**	152.3	68.7	4152.7	4.2	2.2	65.2	Valid
**4**	125.8	38.7	6643.9	3.5	1.1	104.5	Valid
**5**	144.8	73.0	4064.2	4.0	2.1	63.8	Invalid
**6**	133.1	62.6	6552.2	3.7	1.7	103.0	Invalid
**7**	141.9	38.9	8022.6	3.9	1.2	141.7	Valid
**8**	177.0	70.4	5959.4	4.8	2.2	110.3	Valid
**9**	250.0	173.8	8832.9	6.5	4.7	139.5	Invalid
**10**	320.8	245.1	6940.3	8.2	6.6	116.4	Invalid
**11**	283.7	199.9	6736.7	7.3	5.5	129.6	Invalid
**12**	322.8	220.4	10873.6	8.2	6.3	179.4	Invalid
**13**	303.4	201.4	8743.9	7.8	5.7	144.4	Invalid
**14**	296.2	206.1	6409.1	7.6	5.8	94.9	Invalid
**15**	311.3	237.7	6416.8	7.9	6.3	132.4	Invalid
**16**	274.0	199.3	10232.9	7.0	5.3	161.6	Invalid
**17**	283.3	195.0	8968.9	7.3	5.5	142.2	Invalid
**18**	298.6	222.5	6411.6	7.6	6.1	106.8	Invalid
**19**	285.2	188.8	5881.3	7.3	5.4	108.7	Invalid
**20**	242.0	171.5	10098.4	6.3	4.5	158.9	Invalid
**21**	343.6	228.2	11083.1	8.8	6.6	179.1	Invalid
**22**	335.7	259.8	6943.5	8.5	6.8	131.5	Invalid
**23**	315.8	226.4	6939.6	8.1	6.3	104.5	Invalid
**24**	311.5	222.7	11561.8	8.0	6.1	187.0	Invalid
**25**	292.6	211.7	8045.2	7.5	5.6	159.2	Invalid
**26**	269.0	175.6	8806.6	6.9	4.9	138.5	Invalid
**Original**	132.5	55.8	5396.6	3.7	1.6	84.8	
**Min**	125.8	38.7	4064.2	3.5	1.1	63.8	
**Max**	343.6	259.8	11561.8	8.8	6.8	187.0	
**STD**	75.4	74.4	2026.5	1.8	1.98	32.8	

Table 9a. Graphic representation of how similar they are to the original values obtained from the enhanced extruded FE models (percentually)

	Strain			Stress			Validity
	Mean	Median	Max	Mean	Median	Max	
**1**	116.51	98.37	123.93	114.25	103.84	146.61	Valid
**2**	98.54	118.53	98.32	99.15	108.98	98.27	Invalid
**3**	114.96	123.04	76.95	113.19	131.59	76.91	Valid
**4**	94.96	69.40	123.11	95.02	65.17	123.17	Valid
**5**	109.30	130.75	75.31	108.63	127.90	75.24	Invalid
**6**	100.44	112.11	121.41	99.77	103.76	121.41	Invalid
**7**	107.13	69.60	148.66	105.48	72.63	167.07	Valid
**8**	133.58	126.13	110.43	129.02	133.29	130.00	Valid
**9**	188.70	311.28	163.67	175.60	287.73	164.42	Invalid
**10**	242.09	439.15	128.60	221.56	400.10	137.23	Invalid
**11**	214.09	358.07	124.83	197.19	333.69	152.80	Invalid
**12**	243.59	394.76	201.49	223.92	382.55	211.56	Invalid
**13**	228.98	360.71	162.03	211.22	347.43	170.25	Invalid
**14**	223.55	369.15	118.76	205.86	350.61	111.88	Invalid
**15**	234.95	425.86	118.90	214.96	380.50	156.07	Invalid
**16**	206.78	357.11	189.62	191.30	320.92	190.50	Invalid
**17**	213.83	349.36	166.19	197.62	331.57	167.64	Invalid
**18**	225.37	398.56	118.81	206.86	367.71	125.90	Invalid
**19**	215.24	338.28	108.98	198.99	324.93	128.20	Invalid
**20**	182.65	307.14	187.12	170.27	273.68	187.33	Invalid
**21**	259.30	408.79	205.37	237.76	398.00	211.18	Invalid
**22**	253.34	465.45	128.66	231.38	413.76	155.00	Invalid
**23**	238.31	405.58	128.59	218.51	380.80	123.22	Invalid
**24**	235.06	398.96	214.24	216.56	368.47	220.43	Invalid
**25**	220.86	379.23	149.08	202.54	339.29	187.64	Invalid
**26**	203.05	314.62	163.19	188.47	299.12	163.30	Invalid

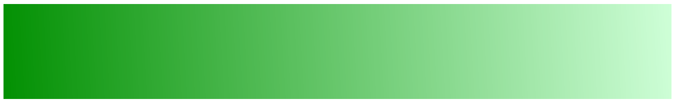

Supplement: Supplementary Information [file rsif20190674supp1.pdf]
